# Citizen-science reveals changes in the oral microbiome in Spain through age and lifestyle factors

**DOI:** 10.1038/s41522-022-00279-y

**Published:** 2022-05-19

**Authors:** Jesse R. Willis, Ester Saus, Susana Iraola-Guzmán, Ewa Ksiezopolska, Luca Cozzuto, Luis A. Bejarano, Nuria Andreu-Somavilla, Miriam Alloza-Trabado, Andrea Blanco, Anna Puig-Sola, Elisabetta Broglio, Carlo Carolis, Julia Ponomarenko, Jochen Hecht, Toni Gabaldón

**Affiliations:** 1 Centre for Genomic Regulation (CRG), The Barcelona Institute of Science and Technology, Dr. Aiguader, 88, 08004 Barcelona, Spain; 2 Life Sciences Programme. Barcelona Supercomputing Centre (BSC-CNS) Jordi Girona, 29, 08034 Barcelona, Spain; 3 Mechanisms of Disease Programme. Institute for Research in Biomedicine (IRB), The Barcelona Institute of Science and Technology, Baldiri Reixac, 10, 08028 Barcelona, Spain; 4 Experimental and Health Sciences Department. Universitat Pompeu Fabra (UPF), Dr. Aiguader, 88, 08004 Barcelona, Spain; 5 Catalan Institution for Research and Advanced Studies (ICREA), Barcelona, Spain; 6 Centro de Investigación Biomédica En Red de Enfermedades Infecciosas (CIBERINFEC), Barcelona, Spain

**Keywords:** Microbiome, Next-generation sequencing

## Abstract

The relevance of the human oral microbiome to our understanding of human health has grown in recent years as microbiome studies continue to develop. Given the links of the oral cavity with the digestive, respiratory and circulatory systems, the composition of the oral microbiome is relevant beyond just oral health, impacting systemic processes across the body. However, we still have a very limited understanding about intrinsic and extrinsic factors that shape the composition of the healthy oral microbiome. Here, we followed a citizen-science approach to assess the relative impact on the oral microbiome of selected biological, social, and lifestyle factors in 1648 Spanish individuals. We found that the oral microbiome changes across age, with middle ages showing a more homogeneous composition, and older ages showing more diverse microbiomes with increased representation of typically low abundance taxa. By measuring differences within and between groups of individuals sharing a given parameter, we were able to assess the relative impact of different factors in driving specific microbial compositions. Chronic health disorders present in the analyzed population were the most impactful factors, followed by smoking and the presence of yeasts in the oral cavity. Finally, we corroborate findings in the literature that relatives tend to have more similar oral microbiomes, and show for the first time a similar effect for classmates. Multiple intrinsic and extrinsic factors jointly shape the oral microbiome. Comparative analysis of metabarcoding data from a large sample set allows us to disentangle the individual effects.

## Introduction

The oral cavity is inhabited by an abundant and diverse microbial community, the oral microbiome, which has been related to processes relevant for health and disease^1^. The mouth is highly vascularized^2^, and is an entry point to the respiratory and digestive systems. As a result, changes in the composition of the oral microbiome can reflect and/or influence systemic changes across the human body, and as such it has an important diagnostic and therapeutic potential. A multitude of factors, both intrinsic (e.g., pH, immune system, chronic disorders) and extrinsic (e.g., lifestyle, diet), have the potential to shape the oral microbiome, but these are as yet only poorly understood. Increasing our knowledge on how these factors alter the oral microbiome is important for unveiling the specific roles that certain oral microbes play in disease processes, which in turn may pave the way for the development of innovative microbiome-based diagnostic and therapeutic approaches.

Most studies on the oral microbiome have focused on delineating its changes in the context of common oral diseases such as periodontitis, gingivitis, or dental caries^3,4^. In recent years, however, the relationships of the oral microbiome with systemic diseases or chronic disorders have received growing attention. These include, among others, different cancer types^5,6^, cardiovascular diseases^7,8^, diabetes^9^, celiac disease^10–12^, Down Syndrome (DS)^13^, or cystic fibrosis (CF)^14^. Thanks to these studies, we are beginning to understand how oral or systemic disorders relate to changes in the composition of the oral microbiome. However, given the strong focus on disease, we still lack a sufficient understanding of non-disease parameters that shape the healthy oral microbiome. These intrinsic (host biology) or extrinsic (environment, lifestyle) factors are pervasive and likely influence not only the overall composition of the oral microbial ecosystem, but also how it will respond in the context of disease, perhaps predisposing one to either relative dysbiosis or resilience.

A relevant intrinsic factor that has been poorly studied in relation to the oral microbiome is age. To our knowledge, there are no studies using high throughput sequencing techniques which focus specifically on the effects of aging on the oral microbiome in a state of relative health and which include a representative spectrum of ages. Recent reviews that have explored aging largely highlight the tendency toward increased periodontitis and dental caries, but they rely primarily on studies using culture-based identification techniques in regards to alterations in particular taxa^15–17^. Some studies which have compared age groups have some limitations, such as narrow age ranges or a focus on age only in the context of particular diseases^15,18–20^. Nonetheless, there are conjectures throughout the literature in reference to the oral microbiome’s role in, and impact from, the physiological changes that occur during the human aging process. Perhaps most notable is the chronic low-grade systemic inflammation sometimes called “inflammaging”, which coincides with immunosenescence, wherein the adaptive immune system declines and the efficiency of innate immunity diminishes with age^17,21^. Thus, further investigation into the connections between age and the oral microbiome is warranted.

Lifestyle and hygiene are perhaps the most studied extrinsic factors with respect to changes in the oral microbiome^22,23^. Smoking^24–27^, wearing braces^28–31^, and the composition of drinking water^28,32^ are factors that have been shown to drive particular changes in the oral microbiota. Extrinsic variables like these impact the oral microbial composition, and in fact, multiple studies have demonstrated that lifestyle, social structures, and shared environments are generally more significant than intrinsic factors like the human hosts’ genetics. Family members have been shown to display more similar microbiome compositions to each other than to non-family members, while there was not a greater similarity amongst monozygotic twins than amongst dizygotic twins^20,33–36^.

Bacteria have received most of the attention in microbiome studies, but other organisms like fungi are also important components. In the oral cavity, species like *Candida albicans* have been implicated in dental caries^37^, wherein it can adhere to the biofilms of the bacterial species *Streptococcus mutans* and both can act to demineralize tooth enamel^38,39^. One study showed two distinct mycotypes (clusters of samples based on the fungal composition), with one being dominated by *Candida* species, and the other with higher fungal diversity and *Malassezia* as the main genus^40^. This and another study^41^ distinguished associations with bacterial taxa in *Candida*-dominated versus other samples, though those results do not seem to coincide entirely. The interactions between bacteria and fungi are an interesting aspect of the oral microbiome that deserves greater attention.

Here, we have taken advantage of the second edition of a large-scale citizen science-based project called “Saca La Lengua” (SLL2 - “Stick Out Your Tongue” in English)^28,42^ to explore the effects of some of these factors in the oral microbiome. Citizen science has been defined by the European Citizen Science association as “an approach that actively involves citizens in scientific endeavor that generates new knowledge or understanding”^43^. Contrary to disease-focused studies, studies on the overall population enabled by citizen-science provide a unique opportunity to infer the effects of commonly present factors. The dataset comprises 1648 oral rinse samples taken from locations across Spain, representing a broad and balanced range of ages. A subset of the samples were from individuals with chronic disorders that are relevant to the physiology of the oral cavity, and all participants filled out a comprehensive survey with questions about lifestyle, diet, and hygiene habits. We coupled this information with 16 S rRNA metabarcoding, as well as culture and proteomics-based identification of fungi to study some of the influences on and of the oral microbiome.

## Results

### Oral microbiome changes through age

**Fig. 1:**
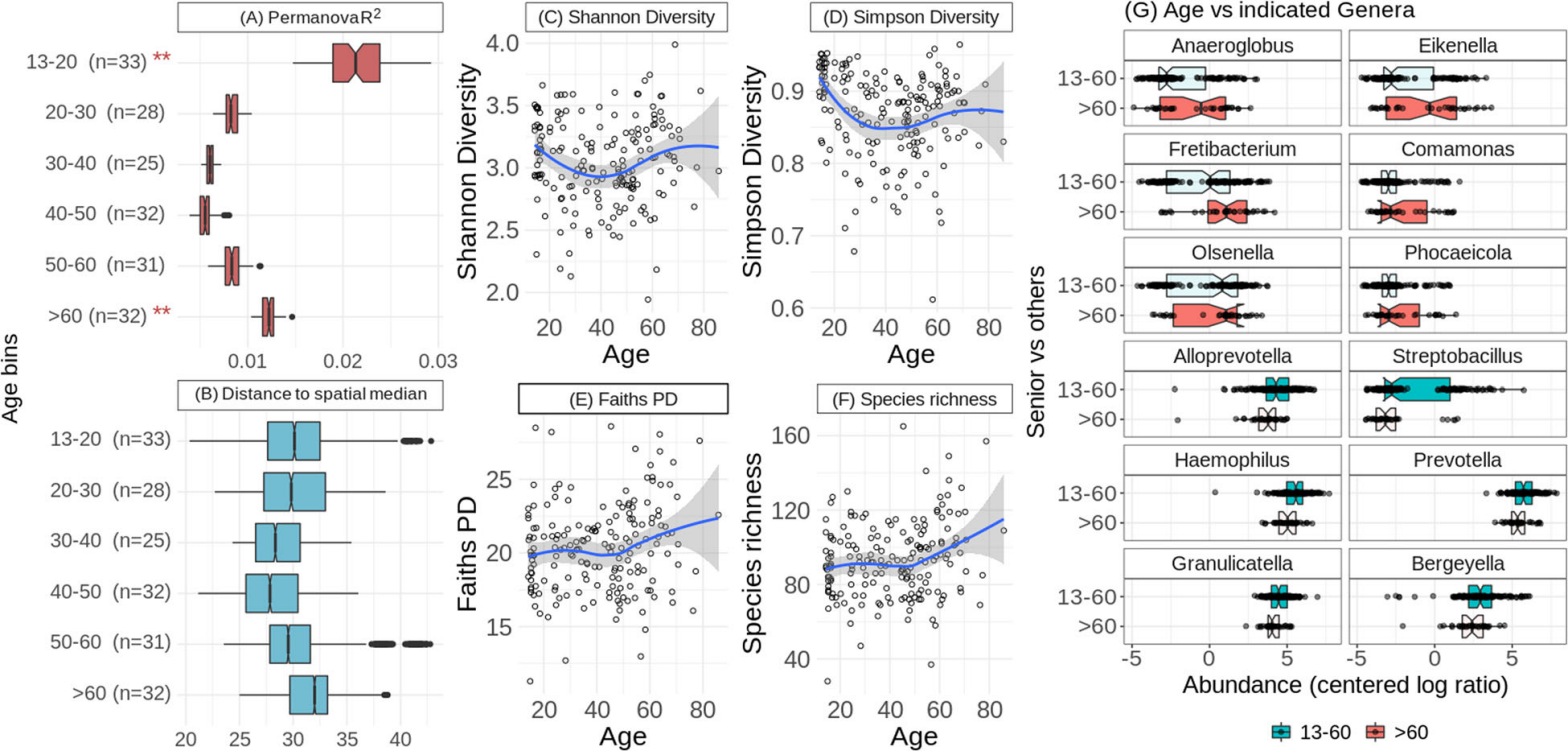
Homogeneity, distinction of composition, and alpha diversity across age. **A** Boxes of the R² values are from the PERMANOVA tests run separately for each of the 100 subsamples. The *n* in both plots indicates the number of samples in a given age bin in each subsample. Red stars indicate the magnitude of the mean adjusted p-values for the PERMANOVA tests. The representation of *p*-values are represented with symbols as indicated in the following value intervals: 0 “***” 0.001 “**” 0.01 “*” 0.05, Not significant. **B** Boxes for the distances to the spatial median represent those distances of each sample from the spatial median of its particular age bin, as calculated by the *betadisper* function. The spatial medians for age bins and the associated ANOVAs were run separately for each of the 100 subsamples, but the boxes here display all such distances for each age bin. **C–F** Tests of the four alpha diversity measures (Shannon, Simpson, Faith’s PD, species richness) were also run with those same 100 subsamples, using age as a continuous value, and the statistical values are summarized in Table 1. The four respective scatter plots here display only the values from one of those subsamples to give a representative depiction of the trend (the same subsample is used for all four), with age (in years) along the *x*-axis. **G** Genera that increase with age tend to be found at lower abundances while those that decrease with age tend to have greater abundances. Boxes display the distributions of abundances of genera (samples were divided into two age groups merely to generalize the tendencies across age: 13–60 years old, or older than 60). The first six genera were those that increased with age (the red boxes for >60 year old individuals are highlighted), and these have notably low abundances in general, while the latter six genera, which decreased with age (the blue boxes for 13–60 year old individuals are highlighted), tend to be found at high abundance, with the exception of *Streptobacillus*, which is more variable.

To assess the impact of aging on the oral microbiome, we compared the microbial profiles of oral rinse samples across ages, using a subsampling strategy that ensures comparable sample sizes (see Materials and Methods). We first tested for changes in the overall microbiome composition across age, including gender and population as fixed effects in 100 such subsamples (see Materials and Methods). PERMANOVA tests based on an Aitchison distance matrix considering age as a continuous value were consistently significant (mean adjusted *P* = 0.001, mean R^2^ = 0.023, mean F statistic = 4.37). To further explore the age ranges that were most distinct, we also used age as a categorical variable, subsampling from the following age bins so that each bin had 25–33 samples: 13–20 years old (964 total samples), 20–30 (41 total samples), 30–40 (28 total samples), 40–50 (85 total samples), 50–60 (46 total samples), >60 (42 total samples), which also showed significant differences (mean adjusted R = 0.001, mean R^2^ = 0.051, mean F statistic = 1.90). Interestingly, when comparing each age bin separately against the group of all others, we observed a sort of parabolic effect, where only those comparisons of the extreme bins (13–20 and >60) against all other bins had a significant result on average across the 100 subsamples, and the differences involving the intermediate bins (30–40 and 40–50) were not significant (Fig. 1A). We further calculated the homogeneity in the microbial composition of samples within a given bin. This homogeneity test first calculates a spatial median for each age bin (a sort of hypothetical centroid composition of the samples within a given age bin, derived from an Aitchison distance matrix), then calculates the distance of each sample in that bin to the spatial median. This test resulted in a similar parabolic effect to that of the PERMANOVA tests (mean adjusted ANOVA *P* = 0.0016, mean F = 4.85), wherein the 40–50 bin was the most homogeneous in terms of microbiome composition, and the >60 bin was the most variable (Fig. 1B).

We performed the same PERMANOVA and homogeneity tests to explore age bins based on both weighted and unweighted UniFrac distances (Supplementary Fig. 1). The PERMANOVA tests were significant for both the weighted UniFrac distance (mean adjusted *P* = 0.006, mean R^^2^ = 0.115, mean F statistic = 4.58) and the unweighted UniFrac distance (mean adjusted *P* = 0.001, mean R^^2^ = 0.063, mean F statistic = 2.37). When comparing each age bin separately against the group of all others, for the weighted UniFrac distance, the youngest bin (13–20) was the only bin with a significant result on average across the 100 subsamples (Supplementary Fig. 1A), while for the unweighted UniFrac distance both the youngest (13–20) and oldest (>60) bins were significant (Supplementary Fig. 1B). The results of the homogeneity test were not significant for either the weighted UniFrac (mean adjusted ANOVA *P* = 0.549, mean F = 1.41, Supplementary Fig. 1C) or the unweighted UniFrac (mean adjusted ANOVA *P* = 0.583, mean F = 1.33, Supplementary Fig. 1D) distances.

In addition, some alpha diversity measures showed parabolic relationships with age, wherein Shannon and Simpson diversity values were lower in the middle ages, consistent with the above result that these were the most homogeneous samples, while Faith’s phylogenetic diversity (PD) and species richness each increased with age, especially in older individuals, though with less statistical significance than the Shannon and Simpson diversities (Fig. 1C–F, Table 1). In Table 1, the *p*-values from ANOVA tests for both quadratic and linear models for these alpha diversity values are displayed, showing that indeed the quadratic model better explains the trends across age.

**Table 1:** Significance of differentially abundant taxa and alpha diversity measures as age increases.

Tax level/variable	Organism/value	Across age	Mean adj. *P*	# of sig. tests
Genus	*Anaeroglobus*	↗	0.0004	100
	*Eikenella*	↗	0.0033	100
	*Fretibacterium*	↗	0.0013	99
	*Comamonas*	↗	0.02	92
	*Olsenella*	↗	0.028	87
	*Phocaeicola*	↗	0.037	75
	*Alloprevotella*	↘	0.0003	100
	*Streptobacillus*	↘	0.0026	100
	*Haemophilus*	↘	0.0072	98
	*Prevotella*	↘	0.016	93
	*Granulicatella*	↘	0.02	93
	*Bergeyella*	↘	0.035	83
Phylum	Synergistetes	↗	0.0002	100
	Bacteroidetes	↘	< 0.0001	100
	Proteobacteria	↘	0.031	80
Physiology	BMI	↗	< 0.0001	100
	pH	↘	0.0026	100
Alpha Diversity	Simpson’s diversity	↘ - ↗	Q = 0.0031 L = 0.26	Q = 100 L = 0
	Shannon’s diversity	↘ - ↗	Q = 0.021 L = 0.99	Q = 90 L = 0
	Species Richness	➙ - ↗	Q = 0.04 L = 0.071	Q = 83 L = 52
	Faith’s PD	➙ - ↗	Q = 0.076 L = 0.12	Q = 0 L = 9

Columns indicate, in this order, the taxonomic level or type of variable, the organism name or variable name, the tendency of the change across age (“**↗”**: increases with age, “**↘”**: decreases with age, “↘ - **↗”**: parabolic effect seen in age, “➙ **- ↗”**: steady across most ages with an increase particularly in older samples), the mean adjusted *p*-value from the ANOVA of the generalized linear or quadratic model, and the number of subsamples for which the test is significant. Rows are ordered first by the tendency with age, with organisms/variables that increase first, and then by mean adjusted *p*-value. In the last two columns for the alpha diversity measures, values are displayed for models based on both quadratic functions of age (Q) and linear functions of age (L).

We next investigated which organisms show significant differences across age. Our results (Table 1) show a number of taxa that increase with age, including the genera *Anaeroglobus*, *Eikenella*, *Fretibacterium*, *Comamonas*, *Olsenella*, and *Phocaeicola*, as well as the phylum Synergistetes, or decrease with age, including the genera *Alloprevotella*, *Streptobacillus*, *Haemophilus*, *Prevotella*, *Granulicatella*, and *Bergeyella*, as well as the phyla Bacteroidetes and Proteobacteria. Of note, genera that increase with age are typically found at low abundance among all samples, whereas those that decrease with age tend to display the opposite trend (Fig. 1G). There was also a marked decrease in pH and increase in BMI as age increased (Table 1).

### Chronic disorders, smoking, and the presence of yeasts in the oral cavity, are important drivers of the oral microbiome composition

**Fig. 2:**
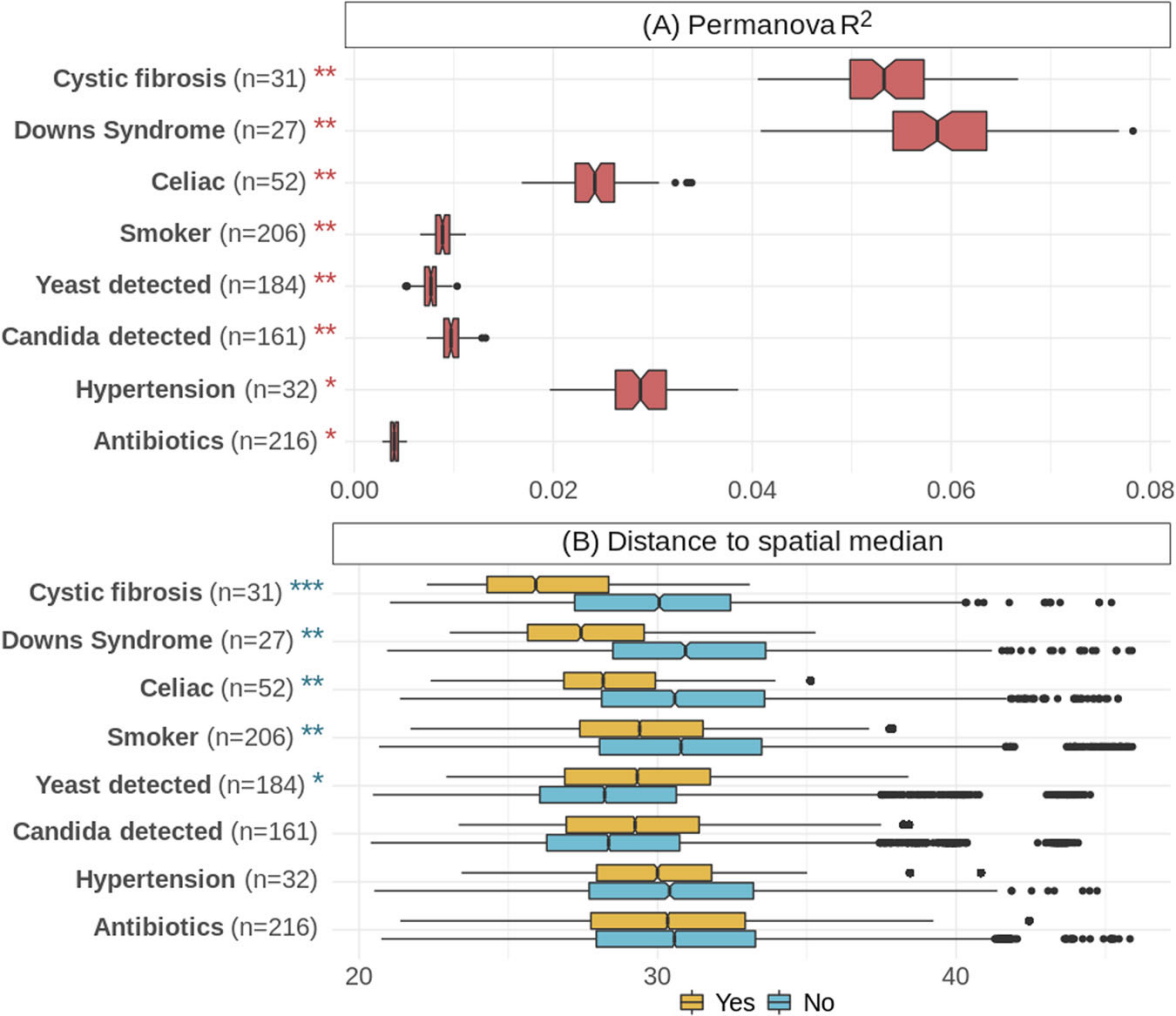
Homogeneity and distinction of composition across variables. **A** Boxes show the distribution of R^2^ values (the proportion of sum of squares from the total) from the PERMANOVA tests comparing groups of a given variable for the 100 subsamples. **B** Boxes represent the distances of each sample from the spatial median of its group (Yes in yellow, No in blue), as calculated by the *betadisper* function. The spatial medians for groups and the associated ANOVAs were run separately for each of the 100 subsamples, but the boxes here display all such distances for each group. Pairs of boxes in both plots are ordered by the absolute value of the difference between the pairs. The n in both plots indicates the number of samples for which a given variable was indicated (the same number of matched controls were selected for each subsample test). Red stars in (**A**) and blue stars in (**B**) indicate the magnitude of the mean adjusted *p*-values for the PERMANOVA tests and the ANOVAs of the betadisper tests, respectively. The representation of *p*-values are as follows: 0 “***” 0.001 “**” 0.01 “*” 0.05, Not significant.

We collected a comprehensive questionnaire regarding over 80 aspects of lifestyle, diet, hygiene, and health from all of the 1648 participants in this study. To assess which of the considered variables had the largest effects on the overall composition of the oral microbiome, we used a PERMANOVA test for each variable with an Aitchison distance matrix, including age, gender, and population as fixed effects (see Materials and Methods). For each of the tested variables, 100 subsamples were taken to match the groups in that variable (Yes vs No) by geographic location, age, and gender. In these comparisons, we excluded samples from donors with any reported chronic disorders, except when the variable of interest was such a disorder. Our results (Fig. 2A) show that chronic disorders like CF (mean adjusted *P* = 0.0011, mean R^2^ = 0.054, mean F statistic = 3.39) and DS (mean adjusted *P* = 0.0013, mean R^2^ = 0.059, mean F statistic = 3.33) were the variables that were most distinct between groups. The detection of yeast species in a sample (as well as the detection of *Candida* specifically), smoking, celiac disease, hypertension, and the reported use of antibiotics also had significant (mean adjusted *P* < 0.05) PERMANOVA results. We could corroborate the general magnitudes of these effects using a multinomial test which included each of the variables mentioned here, as well as age as a continuous value (Supplementary Fig. 2) and age bins as described in the previous section (Supplementary Fig. 3).

A test of homogeneity of each variable showed significant differences when compared to the respective matched controls for CF, DS, the presence of yeast, smoking, and celiac disease (Fig. 2B). As described above with age groups, the result of this test indicates that the samples of one group for a given variable (e.g., those with CF) were significantly more similar to their median composition than the samples of the other group for that variable (e.g., those without CF) were to their own median composition. Interestingly, those samples in which yeasts were not detected were more homogeneous than those in which yeasts were detected. Meanwhile the individuals with CF, DS, and celiac disease, as well as smokers were more homogeneous than those without these disorders and non-smokers, respectively. There was no difference in homogeneity based on hypertension, the use of antibiotics, or the presence of *Candida*, though as with the general detection of yeast, the absence of *Candida* did tend to present greater homogeneity. We included only *Candida* specifically here because it makes up the majority of the yeasts that were detected (present in 236 of 264 samples in which yeast was detected) and no other genus of yeast appeared in more than eight samples.

Some of these variables displayed particular significant differences when compared to their matched controls (Table 2). CF^14^ and DS^13^ have been explored in detail elsewhere, and so are not included in this table. Celiac samples had higher abundances of the genera *Phocaeicola* and *Staphylococcus*, and also had lower Faith’s PD values and species richness (the number of species detected in a sample). Smokers had higher abundances of *Megasphaera*, *Fretibacterium*, and *Streptococcus*, and lower abundances of *Fusobacterium*, *Capnocytophaga*, *Bergeyella*, *Porphyromonas*, *Leptotrichia*, *Haemophilus*, *Neisseria*, *Lautropia*, and an unclassified genus of the class Gracilibacteria, and also had lower Simpson and Shannon alpha diversity values. Samples in which yeast were detected, in particular those with *Candida*, had higher abundance of *Lactobacillus*. There were no individual taxa that differed significantly for hypertension or antibiotics.

**Table 2:** Significance of differentially abundant taxa and alpha diversity measures between indicated variable and matched controls.

Sample group	Organism/diversity	Tendency	Mean adj. *P*	# of sig tests
Celiac	*Phocaeicola*	↗	0.08	63
	*Staphylococcus*	↗	0.09	50
	Faith’s PD	↘	0.0009	100
	Species richness	↘	0.0004	100
Smokers	*Megasphaera*	↗	0.0017	100
	*Fretibacterium*	↗	0.037	77
	*Streptococcus*	↗	0.07	66
	Phylum: Synergistetes	↗	0.003	100
	Phylum: Firmicutes	↗	0.042	76
	*Fusobacterium*	↘	0.0003	100
	*Capnocytophaga*	↘	0.0004	100
	*Bergeyella*	↘	0.0028	100
	*Porphyromonas*	↘	0.018	89
	*Leptotrichia*	↘	0.022	88
	*Haemophilus*	↘	0.03	83
	*Neisseria*	↘	0.031	77
	*Lautropia*	↘	0.051	65
	C.Gracilibacteria.UCG	↘	0.056	70
	Phylum: Fusobacteria	↘	0.0016	100
	Phylum: Patescibacteria	↘	0.025	93
	Simpson diversity	↘	0.002	100
	Shannon diversity	↘	0.029	83
Yeast detected	*Lactobacillus*	↗	0.053	61
*Candida* detected	*Lactobacillus*	↗	0.01	96

Columns indicate, in this order, the variable considered, the organism name or the alpha diversity value, the tendency of the difference in the considered variable (“**↗”**: higher in those samples where the variable is true, “**↘”**: lower), the mean adjusted *p*-value of the ANOVA of the statistical comparison between variable and matched controls, and the numbers of matched control subsamples for which the test is significant. Rows are ordered first by the tendency in the indicated variable, with organisms/diversities that were greater first, and then by mean adjusted *p*-value within each variable group.

Co-occurrence networks represent patterns of taxa that present correlated abundances across different samples^44^. We constructed such networks for groups of samples differing in the studied variables and compared them in the search of unique associations between taxa. From these network comparisons, we derived a score that indicates the relative network uniqueness (i.e., the fraction of significant co-occurrences that are unique to that variable—see Materials and Methods) (Supplementary Fig. 4). The most unique co-occurrence networks were seen in samples with CF (the specifics of this network were discussed in a previous publication^14^) and hypertension, followed by the absence of yeast (and specifically the absence of *Candida*), then the other two chronic disorders, DS and celiac, and finally smoking, and the reported use of antibiotics. This largely follows the trend in the PERMANOVA results presented above, wherein the samples that are more distinct from their matched controls largely display the more unique sets of significant co-occurrences, though did not necessarily follow the same pattern as the explained variances from those PERMANOVA tests. DS had greater R² values than hypertension and the detection of yeast, and *Candida* specifically, yet generally had a less unique network than those three variables. Neither did the network uniquenesses show the same trend as the homogeneity results, as hypertension, for instance, showed no difference in homogeneity, yet had the second most unique network, while smoking showed one of the strongest differences in homogeneity, and was the second least unique network.

We performed predictions of the functional content of our oral microbiome samples in different contexts. The only variables found to show significant differences in any KEGG orthologs (KOs) were smoking and DS (Supplementary Fig. 5 shows the only other variables that had p-values from ANOVA tests less than 1.0). Smoking had not only the most KOs that differed significantly (430), but also the strongest differences, as seen in that heatmap. DS had a total of 99 significantly different KOs. Those 430 KOs that differed with smoking and those 99 that differed with DS were associated with 168 and 88 pathways, respectively. However, there were many instances of pathways that were associated with some KOs that were increased in smokers and others that were decreased in smokers, and the same for DS (Supplementary Fig. 6). Nonetheless, we used a text mining approach to search for articles that found links between a given pathway and either smoking or DS, also shown in Supplementary Fig. 6. The most prominent pathways found to be significant here and that are associated with smoking in the literature were carbon metabolism (500 articles), fatty acid metabolism (322), purine metabolism (263), base excision repair (197), and biosynthesis of amino acids (188). For DS, the most prominent were also biosynthesis of amino acids (130 articles), carbon metabolism (28), and oxidative phosphorylation (18).

We found that the pH of the oral cavity was anti-correlated with some measures of alpha diversity, including Faith’s PD (*p* = 2.97e-5) and species richness (*p* = 6.23e-5), while there was no association with either Shannon or Simpson diversity (Supplementary Fig. 7). There were also trends, either positive or negative, with a number of particular genera (Supplementary Table 1).

### Similarity of the oral microbiome composition among family members and classmates

**Fig. 3:**
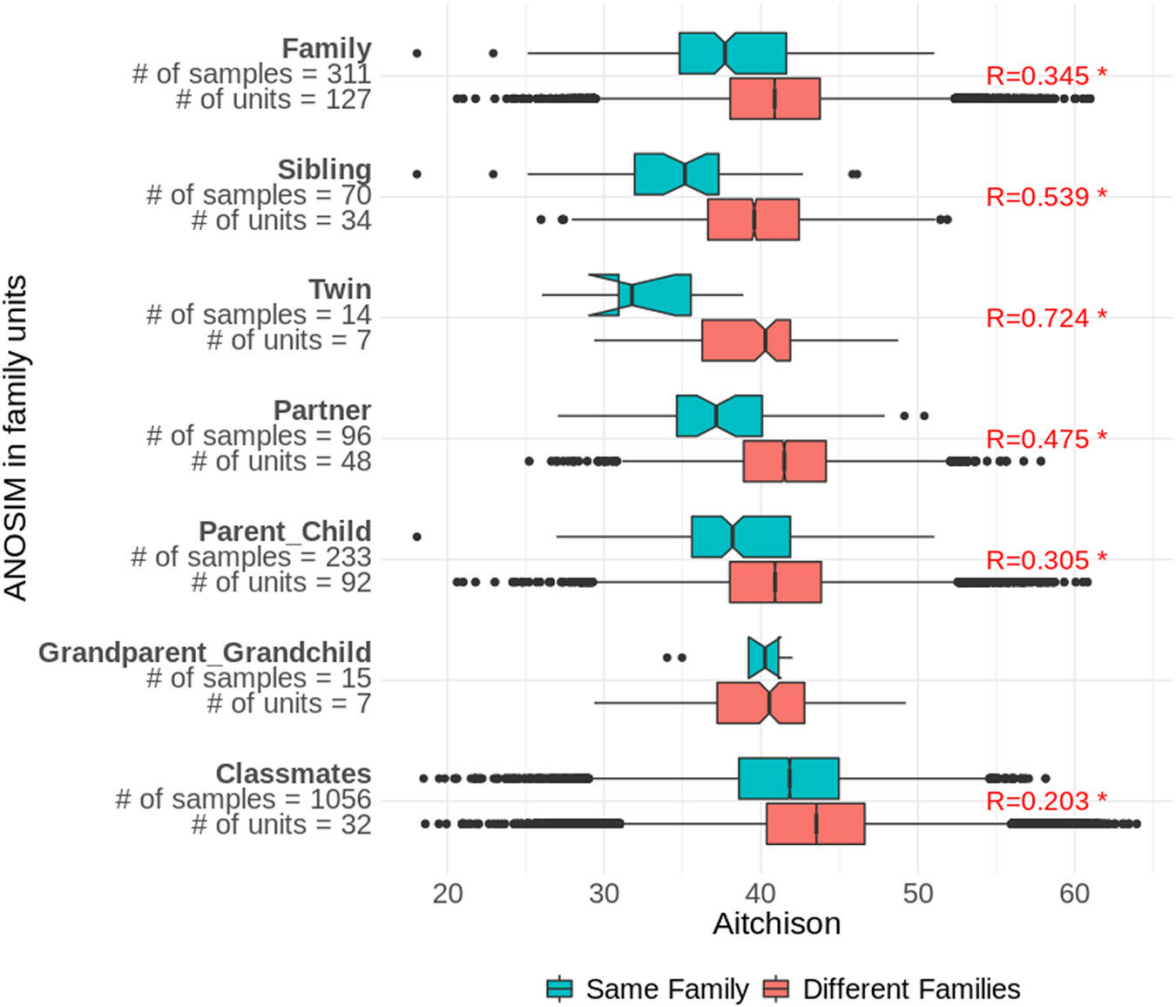
Anosim analyses of family units of various degrees of relationships, as well as classmates. Boxes show the distributions of Aitchison distance values between samples from the same unit (blue) or different units (red). The anosim R statistic is shown for those relationships that had significant results (anosim *P* < 0.05). The *y* axis labels indicate, for each relationship type, the number of samples for which that relationship occurred in at least one other sample, and the number of different units of two or more samples for which that relationship occurred.

Our study included groups of samples that belong to members of the same family, and specified different degrees of relationship, such as parents and children, grandparents and grandchildren, partners, siblings, and twins. In addition, given the active participation of schools in our project, we had several groups of samples from students attending the same school. Using an anosim test (analysis of similarities) on Aitchison distance matrices, we compared the similarity between the microbiome profiles of members of the same family or classroom, to determine whether the similarity was significantly higher than when compared to samples from different families or classes. With the exception of grandparents and grandchildren, all other relationships showed significantly greater similarity in oral microbiome compositions than was seen between samples from other families or classes (Fig. 3). This similarity was highest for twins, followed by siblings, partners, family members (which included all of the non-classmate connections), parents-children, and classmates. Although the anosim statistic was higher for twins than for siblings, that merely indicates that the trend was stronger in twins. But twins were not statistically more significant to each other than siblings were to each other (*P* = 0.33 for the Mann–Whitney test of Aitchison distances among twins vs those distances among non-twin siblings, the values represented by the blue boxes in Fig. 3).

## Discussion

Our study builds on the first edition of the citizen-science project “Saca La Lengua”^28^, which included 1319 samples that were almost exclusively from 13–15 year old students in relative health. This first edition provided a comprehensive snapshot of the oral microbiome composition in adolescents and how it varied with different lifestyle parameters. In this second edition, we targeted a broad age range (7–85) as well as a few particular chronic disorders, namely CF, DS, and celiac disease, in collaboration with relevant local and national patient associations. Participants also completed a comprehensive questionnaire about various daily habits, hygiene, and diet. When collecting samples, we encouraged participants to bring along family members, and in the end, 311 of the 1648 total samples from this second edition of “Saca La Lengua” (SLL2) had some familial connection. To our knowledge, this is the first study to explore differences in the oral microbiome across a range of ages that is both balanced and encompasses most of the full range of the average human life expectancy (in Spain, recent estimates were 86 years for women and 80 for men^45^). We have reported separately on the specific connections of the oral microbiome with DS^13^ and CF^14^, and here we present results based on the full SLL2 dataset. It is important to note that, as these analyses are based on 16 S rRNA gene amplicon sequencing, there is an inherent limitation in the resolution of taxonomic classification, and here the comparisons of particular taxa are performed at the genus level.

### Oral microbiome changes through age

Studies exploring the trajectory of changes across the human lifespan have been limited, either comparing very disparate age groups^46^, a limited age range^19^, or categorizing samples into very wide age ranges that do not effectively represent that entire range^20^. By spanning adolescence to late adulthood, our dataset provides some new insights into the topic. Our results show significant shifts in composition across time, wherein the younger and older samples were the most distinct, and the middle ages appear to represent an intermediate phase in which the oral microbiome is at its most homogeneous. The parabolic trend in homogeneity was matched by the trend in both the Shannon and Simpson alpha diversity metrics, which were both lowest in approximately the 30–50 year old range. We can extrapolate similar results to ours from some of the studies mentioned above. In one, samples from women between the ages of 53 and 81 showed no significant change in alpha diversity^19^, and at these ages, the diversity values in our samples have already risen to a relative plateau. In another study, a citizen-science project much like our own, youth samples (ages 8–16 with a mean age of 10) showed greater alpha diversity than adult samples (ages 20–75 with a mean age of 34)^20^. Though their “adult” group reaches up to age 75, the mean age of 34 suggests a similar result to our own.

Despite the parabolic trends in both alpha diversity and homogeneity across age, we did not find evidence of these patterns in the abundances of any particular organisms. Instead, we saw that with age there were statistically significant decreases in the genera *Alloprevotella*, *Streptobacillus*, *Haemophilus*, *Prevotella*, *Granulicatella*, and *Bergeyella*, and increases in *Anaeroglobus*, *Eikenella*, *Fretibacterium*, *Comamonas*, *Olsenella*, and *Phocaeicola*. As noted above, a typical trend in the aging oral cavity is an increase in the prevalence and severity of periodontal disease^15–17^. With the exception of *Olsenella*, each of the genera that were increased with age in our samples has been associated with periodontitis^47–57^. While we do not have data on salivary flow rate or nitrate levels from our samples, salivary flow rate has also been shown to decrease in the elderly^46,58^, and is proportional to the systemic concentrations of anti-inflammatory nitric oxide (NO)^59^, the local concentrations of immunoglobulins and various molecules important for the mineralization of tooth enamel, and also maintains pH by removing substrates for the microbiota, as well as their acidic byproducts^60^. Indeed, we also found that pH decreased with age in our samples. Thus, future studies which track oral microbiome changes across age along with periodontal health, salivary nitrate levels and systemic NO levels, which result from an enzymatic process in oral commensal bacteria that humans cannot perform themselves^61,62^, and how these combinations relate to inflammaging, would warrant further attention.

A noteworthy observation in the changes across age in our study is that those genera that decreased with age were typically among the most abundant oral taxa, while those that increased were found at relatively low or median abundances (Fig. 1G). We speculate that the elderly oral microbiome may be more susceptible to colonization and establishment of rare opportunistic species whose growth is hindered by the more efficient immune responses in younger oral cavities. This would be in line with hypotheses proposed to explain the higher prevalence of periodontitis through aging^17^, which relate it to different factors, such as the accumulation of tissue damage, weaker immunity, increased adipose tissue (a source of cytokines), decreased anti-immflamatory sex hormones, diminished physical activity, and increased oxidative damage. Some of these factors may also explain the relatively high alpha diversity values in the elderly samples, though not necessarily in the teenaged samples. These instead may be a result of the continually developing microbiome composition, which appears to reach a more stable state in the 30 s and 40 s. It should also be noted that two of the alpha diversity measures we looked at (Faith’s phylogenetic distance and species richness) were only higher in the older samples, and remained consistent up to the ages of approximately 50–55. Thus younger and older microbiomes present higher diversities of somewhat different natures, with the elderly being characterized by a higher number of species (Species richness) and more phylogenetically diverse compositions (Faith’s PD), whereas both extreme age groups present similarly diverse microbiomes in terms of balanced representations of the different taxa (namely Shannon’s and Simpson’s diversity indexes). Other age groups, in comparison, are characterized by less diverse microbiomes, with more clear separations between dominant and minority taxa. The results of the PERMANOVA tests using the two UniFrac distances further support this hypothesis of differing diversities across age (Supplementary Fig. 1A, B). For instance, both weighted and unweighted distances showed strong differences in the youngest age bin (13–20), but only the unweighted distance showed strong differences for the oldest age bin (>60). This would suggest that, while the youngest group shows high variability in both rare and common organisms, the oldest group is strongly affected by rare and low abundance organisms and not the most common and abundant organisms, and that the differentiation of those older samples is driven largely by phylogenetically distinct organisms.

### Chronic disorders, smoking and the presence of yeasts in the oral cavity, are important drivers of the oral microbiome composition

The presence of chronic disorders such as CF and DS, the most impactful factors seen in this dataset, and their particular impacts were described elsewhere^13,14^. Persons with CF, DS, or celiac disease, as well as smokers, had significantly more homogeneous compositions compared to the matched controls without these disorders and non-smokers, respectively. This finding suggests that those three disorders and smoking not only differentiate those samples significantly from their matched controls, but also that the bacterial compositions are shaped in consistently similar directions (i.e., toward a specific signature), while the controls are comparatively more variable. The reverse was the case for the detection of yeast, so that perhaps greater prevalence of these fungi promote a departure from typical bacterial ecosystems. This supports the existence of diverse synergistic and antagonistic ecological interactions between yeasts and bacterial species, and a role of fungi as keystone species in the oral ecosystem. Alternatively, the presence of yeasts might be a consequence of already unbalanced microbiomes, suggesting they are opportunistic colonizers. In both cases, they could be considered as potential biomarkers for altered microbiomes. Finally, hypertension and antibiotics displayed significant differences to their matched controls, but there was no difference in homogeneity, so these factors did not direct the differences in any specific manner, perhaps depending on the specific antibiotic used or the severity of hypertension, for which we do not have specific information.

The particular differences seen in some of these variables here corroborate some findings in the literature. A study found that never-smokers and former smokers did not differ from each other in composition, but both differed significantly from current smokers, and that smokers had higher *Streptococcus* and *Atopobium*, and lower *Capnocytophaga*, *Leptotrichia*, and *Peptostreptococcus*^24^. We found the same for *Streptococcus*, *Capnocytophaga*, and *Leptotrichia*. Three studies found smokers had increased *Megasphaera* and decreased *Neisseria*^25–27^, though one of those^25^ reported the family *Veillonellaceae*, of which *Megasphaera* is a member. There was also agreement with our finding of a decrease in *Haemophilus*^26,27^, *Lautropia*, *Fusobacterium*, and *Leptotrichia*^27^, though depending on the study, there were opposite findings for *Fusobacterium*, *Streptococcus*, and *Porphyromonas*. A study that described two distinct oral mycotypes (sample clusters defined by the fungal composition), found that one of these was dominated by *Candida*, and was enriched in *Lactobacillus* and *Propionibacterium*^40^, the former of which matches our own finding here. If their reported mycotypes are indeed ubiquitous structures of fungal composition, it may be that our samples also follow this dichotomy and the non-*Candida* samples would perhaps fall in the other mycotype, which was much more diverse in fungi, though this would require further investigation.

Although the relative scores of uniqueness of the co-occurrence networks of the different variables mentioned here did not precisely match the patterns from either the PERMANOVA or homogeneity tests, the unique co-occurrences among particular sample groups suggest underlying ecological differences present under the various conditions. The networks of CF, for instance, were discussed at length elsewhere^14^. Moreover, there was greater variation in the uniqueness scores for hypertension and absence of yeasts/*Candida* than in the other variables, as can be seen in Supplementary Fig. 4B, and thus a greater proportion of the associations in these networks were also seen in the networks of other variables. CF, as a contrasting example, had relatively little variation, and thus consistently displayed many of the same associations that did not appear in the networks of other variables, so its network is more universally unique. Similarly, although the uniqueness score for smokers was relatively low, it also had low variation, so the relatively few unique associations were also universally unique. The caveat to these findings is that here we only compare the networks of those eight variables, which we found to significantly differentiate individuals from matched controls (as in Fig. 2). To better understand the underlying ecologies, a more expansive comparative exploration of co-occurrence networks in particular cohorts should be performed.

### Similarity of the oral microbiome composition among family members and classmates

Our finding that the oral microbiomes among family members are more similar to each other than to those of non-family members corroborates the trends seen in the literature^20,33–35^. One of these studies found that twins were not more similar to each other than non-twin siblings^20^, which we have corroborated in our results here, and another found that monozygotic twins were not more similar to each other than dizygotic twins^33^, which was also seen in the gut microbiome^36^. Moreover, a study using a genome-wide analysis of SNPs to compare genetic similarity with microbiome composition found no significant association^34^. All of this evidence points to the conclusion that the shared environment of the home strongly influences oral microbiome composition, more so than host genetics. In agreement with this, the only familial relationship that did not show a significant similarity in our data was that of the grandparent and grandchild, which is the connection least likely to share a living space. Indeed, while twins had the highest similarity score, they were not significantly more similar to each other than non-twin siblings, further supporting the findings in the literature. We even saw that, among the teenage samples obtained from different high schools, the oral microbiomes were more similar among classmates than non-classmates, though this was the comparison with the lowest magnitude of similarity among those that were significant (lowest anosim R statistic), as would be expected since it generally entails more distanced interactions than those among family members. The result about classmates may suggest that a regularly shared environment, even if only for a few hours a day, could impact the oral microbiome composition. Future studies could explore this notion further, for instance focusing on workplaces with close physical proximity like shared offices in contrast to more distanced outdoor working groups, as in construction sites.

### Citizen-science reveals the relative impacts of important factors shaping the composition of the oral microbiome

This second edition of the citizen-science project Saca La Lengua (SLL2) extends the results of the first edition^28^, which provided a snapshot of the oral microbiome of teenagers in relative health across Spain. Here we have displayed the differences that occur across age, wherein a number of genera of bacteria either increase or decrease in abundance, and people in middle ages typically have more homogeneous compositions than teens or seniors, as well as lower alpha diversity, and seniors tend to harbor a greater number of low abundance organisms and a more acidic oral environment. In SLL2 we also compared the general influence of a number of different health and lifestyle factors on the oral microbiome composition. CF and DS were the most impactful in terms of differentiating the composition, and the samples with these chronic disorders were significantly more homogeneous than matched controls, suggesting the disorders tend to direct the composition of the oral microbiome in specific and consistent ways. A similar effect was seen with celiac disease, smoking, and the absence of yeast species, while hypertension and recent use of antibiotics significantly differentiated samples, but did not show a difference in homogeneity. Nonetheless, hypertension, along with CF, displayed more unique associations between bacterial taxa in co-occurrences networks compared to these other variables, suggesting particular underlying ecologies. We also expanded upon findings in the literature that shared environments are important in shaping the oral microbiome. We saw that family members that typically live within the same household tend to have significantly more similar compositions compared to non-family members, and that twins are not significantly more similar than non-twin siblings, supporting the idea that the environment, more than host genetics, shape the microbiome. Furthermore, we saw that students in the same school were more similar to each other than those from different schools. This opens a door to further studies of shared spaces, like different working environments, as our finding suggests that regularly sharing the same environment for even a few hours impacts the microbiome. This study describes the manners in which an assortment of factors affect the oral microbiome in the Spanish population. The results lay some groundwork for future studies to expand upon in dedicated cohorts for particular factors, as well as in other populations.

## Methods

### Sample collection

All participants signed an informed consent form allowing the use of their saliva samples for microbiological research. For participants under the age of 18, the consent form was also signed by one of the parents or a legal guardian. This project was approved by the ethics committee of the Barcelona Biomedical Research Park (PRBB). Samples were collected from January to November 2017, and a map of the locations from which they were collected is presented in Supplementary Fig. 8. Participants were asked not to ingest any food or beverage (except water) for 1 h before collecting the sample. All donors received clear indications about the sample collection procedure in person, and the collection of the samples was carried out with the assistance of a researcher involved in the project, following a demonstration. All participants responded to a uniform questionnaire about lifestyle, diet, hygiene, and health. Before collection of the oral rinse, the pH of the saliva was measured using pH test strips (MColorpHast, Merck, range 5.0–10.0; 0.5 accuracy units), the accuracy of which have been previously validated^28^. Saliva samples were collected using a mouthwash as described earlier^28^. Different oral collection methods have been shown not to significantly affect taxonomic classification at the genus level, while the oral rinse method, as used in this study, has been shown to yield greater quantities of genetic material as compared to spitting or passive drooling^63^. In brief, the protocol is as follows: participants rinsed their mouth with 15 mL of sterile phosphate-buffered saline (PBS) solution, for 1 min. Then, they returned the liquid into a 50 mL tube. The samples were then centrifuged at 4500 g for 12 min at room temperature (r.t.) in an Eppendorf 5430 centrifuge equipped with an Eppendorf F-35-6-30 rotor. The supernatant was discarded and the pellets were resuspended with the remaining PBS, transferred to 1.5 ml tubes and centrifuged at 4500 g for an additional 5 min at r.t. using an Eppendorf FA-45-24-11-HS rotor. Supernatants were discarded, and pellets were frozen and stored at −80 °C until further analysis.

The methods used for DNA extraction and 16 S amplicon sequencing, fungal composition analysis, the pre-processing of 16 S rRNA sequence reads and taxonomy assignment, as well as the alpha and beta diversity measures that we employed, were described in previous publications which used the same dataset^13,14^.

### Subsampling for analyses

When running statistical tests for a given variable, we first randomly select representative matched controls 100 times to ensure consistency in the results. In the case of binary variables, such as smoking, where the values are either “yes” or “no”, we randomly selected an equal number of samples from each group, and checked if each group had similar distributions of age, geographic location (based on the autonomous community within Spain from which the sample was collected), and gender. If there were over 100 total samples in both the “yes” and “no” groups for a given variable, 100 of each were selected for each of the 100 subsamplings, otherwise the total number of the smaller group were selected and a matched random selection of the same size from the other group. In the case of age groups, we first classified our samples into six bins of ages: 13–20 (964 samples), 20–30 (41 samples), 30–40 (28 samples), 40–50 (85 samples), 50–60 (46 samples), >60 (42 samples). We then ensured that the six age bins had balanced geographical distributions and genders. The 100 subsamples based on the age bins were also used for calculations with age as a continuous variable, in order to ensure an even distribution of ages, as well as to account for the geographical distributions and genders. In all cases, the number of samples taken from each group within a given variable were consistently the same across all 100 subsamples. For instance, in each of the 100 subsamples based on age bins, the following were the number of samples from each bin: 13–20 years old (33 samples), 20–30 (28 samples), 30–40 (25 samples), 40–50 (32 samples), 50–60 (31 samples), >60 (32 samples). And in this case, the differences in sample numbers between these age bins stems from the availability of samples from the balanced ranges of gender and geographical distribution. In all instances, samples that were excluded from control groups were those that had any reported chronic disorder (311 samples), were missing data regarding age or gender, or that were collected from a region other than those from which the samples with the variable of interest were collected, since control groups were selected to match the range of ages and proportions of gender and geographic locations. Relevant *p*-values mentioned throughout the text are the average from the tests across the 100 subsamplings, corrected with the “fdr” method in the p.adjust function from the base “stats” package (version 3.6.3)^64^, unless otherwise stated.

### Statistical analyses

#### Comparisons of compositions

In order to determine the effects of variables on the composition of the oral microbiome, we performed a permutational multivariate analysis of variance (PERMANOVA) based on Aitchison distance metric using the *adonis* function from the “vegan” R package (version 2.5-6)^65^. The model included the following fixed effects: variable of interest (e.g., smoking or age), gender, age (when it is not the primary variable of interest), and population of the city/town from which the sample came (as a generalized proxy of both location and lifestyle).

We used the *betadisper* function from the “vegan” package to test the homogeneity of group variances within the groups for a given variable to compare, for example, smokers versus non-smokers, or among the six age bins. Using the *anova* function from the “stats” package on the betadisper object, we could obtain a p-value to determine whether there was a significant difference in the homogeneity of the compositions of samples between groups. The betadisper objects also hold the distance of each sample within a group from that group’s spatial median, a measure of the centroid composition for each group, and we use these values to display the differences between groups in boxplots.

We used the *anosim* function from the “vegan” package to perform an analysis of similarities based on the Aitchison distance metric among family members and classmates. The different relationships considered were siblings, twins, partners, parents-children, grandparents-grandchildren, all family members (includes any of the previously mentioned relationships), and classmates (samples from students in the same school). For an anosim test of a given relationship, only those samples which had at least one relationship of that type were included. For example, there were 70 samples that had a sibling that also provided a sample, so the sibling anosim test included those 70 samples, wherein 34 distinct groups of siblings occurred (for any relationship, groups were by necessity of two samples or more).

#### Differential abundance and diversity calculations

Then, to determine differential abundances of taxa and variation in other variables like alpha diversity, oral pH, or the measurements of ions in drinking water, we performed a generalized linear model using the function *glm* from the “stats” package, again using the same fixed effects as for the PERMANOVA test. The abundance values used for these tests were the centered log ratios of the amplicon sequence variant (ASV) counts, as described elsewhere^13,14^. For the alpha diversity measures in relation to age in particular, we further used the *bs* function from the base R package “splines” to treat age as a second order fixed effect, in order to detect a parabolic trend. The *Anova* function from the “car” R package (version 3.0-7)^66^ was used to calculate type-II ANOVA tables, from which *p*-values were taken for each fixed effect in the models. These p-values were corrected for multiple testing with the *p.adjust* function from the “stats” package, using the “fdr” method.

#### Inferred co-occurrence networks

To produce co-occurrence networks within the groups for a given variable, we first filtered out very rare taxa to avoid spurious associations in taxa that do not appear regularly, by using the *filterTaxonMatrix* function from the “seqtime” R package (version 0.1.1)^67^. We retained those taxa that had at least 15 counts in at least 20 samples. Then we calculated the networks for each of the groups in a given variable (for instance, for smokers and for non-smokers) in each of the 100 subsamplings using the *spiec.easi* function from the “SpiecEasi” package (version 1.0.7)^44^, from which we could derive the strengths of the significant associations, both positive and negative, between particular taxa. The chord diagrams that we used to represent the uniqueness of networks for particular variables were produced using the chordDiagram function from the “circlize” package (version 0.4.8)^68^. To calculate the relative uniqueness of networks, we developed a score that is relative to the eight variables that we considered, which were those found to be significant with the PERMANOVA test. The scores were calculated as follows: for each variable, the co-occurrence networks were calculated among each of the 100 subsamples, and we retained those associations which occurred only in the groups of interest (samples with the indicated disorder, smokers, antibiotic users, or those samples in which yeast was absent). Then for each variable, we calculated, pairwise with each other variable, the number of only those associations which occurred in all 100 subsamples and in 0 subsamples of the other variable being compared, weighted by the strengths of those associations that were determined by the *spiec.easi* function.

#### Multinomial tests and biplot visualizations

Multinomial regression was performed using the *multinomial* function from the songbird tool (version 1.0.4)^69^ through Qiime 2^70^. The parameters used which differed from the default values were the following: epochs = 5000, batch-size = 164, num-random-test-examples = 247, differential-prior = 0.2, summary-interval = 1. Biplots were produced with the Qiime 2 view tool, based on the output of the emperor biplot tool (version 1.0.3)^71^.

#### Functional prediction analyses

We used the *t4f* function from the themetagenomics R package (version 1.0.2)^72^ to predict the functional content of the oral microbiome in different contexts. This tool produced abundances of KEGG orthologs and associated pathways, which we then checked for differential abundances based on our metadata variables using linear models in the same manner as described for taxa above and using the same fixed effects. The heatmap of the logs of p-values from these tests was produced using the *d3heatmap* function from the d3heatmap R package (version 0.9.0)^73^. Then we used a text mining approach with the *fetch_pubmed_data* function from the easyPubMed R package (version 2.13)^74^ to search for articles which connected a given variable with pathways that differed significantly.

### Reporting summary

Further information on research design is available in the Nature Research Reporting Summary linked to this article.

## Supplementary information

Supplementary material

Reporting Summary Checklist

## Data Availability

The fastq files for the paired forward and reverse reads of the 16 S rRNA sequencing of the 1648 oral rinse samples used for the analyses in this study (57,221 Mb) were uploaded to the Sequence Read Archive (SRA) with the BioProject accession number PRJNA667146 and can be found here: We also provide a table with the results of MALDI-TOF analyses of fungal composition (44 kb), which can be found here: https://github.com/Gabaldonlab/ngs_public/tree/master/SLL2. The unique and anonymized identifiers for each sample can be found at the beginning of each fastq file, and these correspond to the row names in the fungal composition tables.

## Code availability

The R code used to perform the statistical analyses can be found here: https://github.com/Gabaldonlab/ngs_public/tree/master/SLL2.
