# Supplementary material for: Citizen-science reveals changes in the oral microbiome in Spain through age and lifestyle factors
Source: NPJ Biofilms Microbiomes. 2022 May 19;8:38. doi: 10.1038/s41522-022-00279-y (PMC9117221; doi:10.1038/s41522-022-00279-y)
Supplement: Supplementary file 1 — Supplementary material [file 41522_2022_279_MOESM1_ESM.pdf]

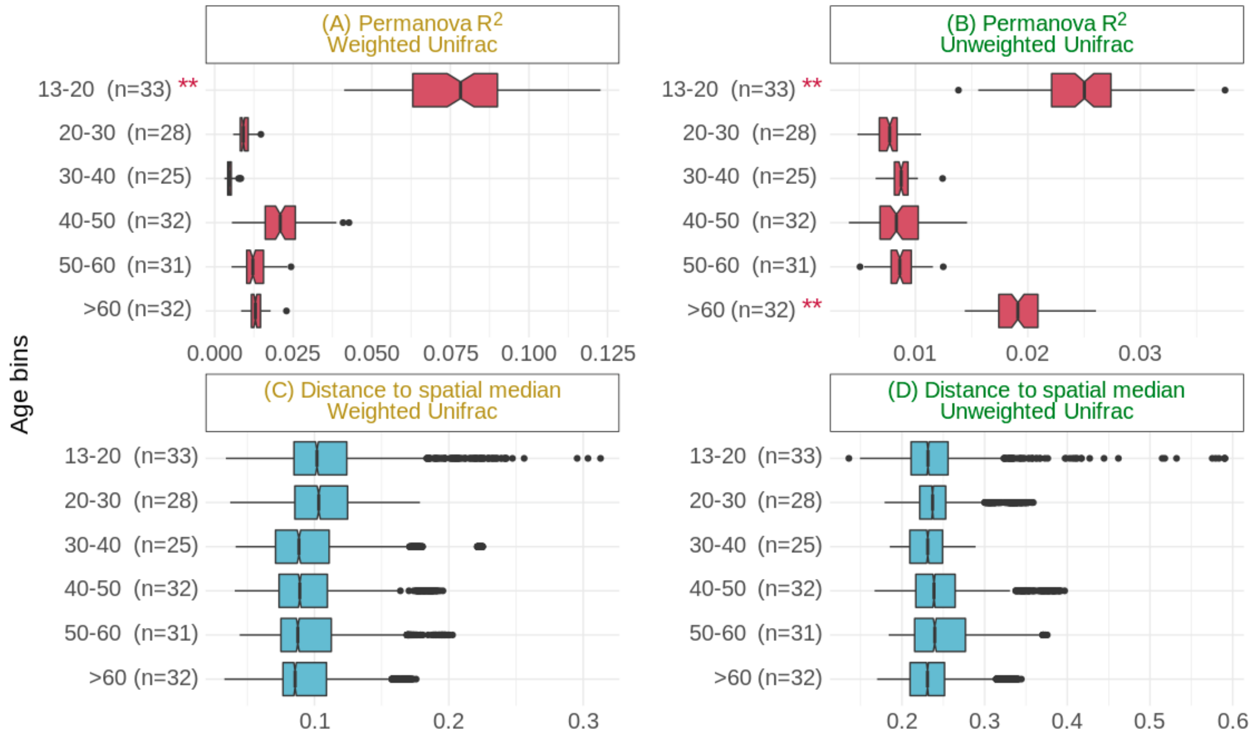

Supplementary Figure 1: Homogeneity and distinction of composition by phylogenetically weighted distance measures. (A-B) Boxes of the R² values are from the PERMANOVA tests run separately for each of the 100 subsamples, based on weighted and unweighted UniFrac distances, respectively. Red stars indicate the magnitude of the mean adjusted p-values for the PERMANOVA tests. **(C-D)** Boxes for the distances to the spatial median represent those distances of each sample from the spatial median of its particular age bin, as calculated by the *betadisper* function, based on weighted and unweighted UniFrac distances, respectively. The spatial medians for age bins and the associated ANOVAs were run separately for each of the 100 subsamples, but the boxes here display all such distances for each age bin. The n in all plots indicates the number of samples in a given age bin in each subsample. The representation of p-values are represented with symbols as indicated in the following value intervals: 0 '***' 0.001 '**' 0.01 '*' 0.05 ' ' Not significant.

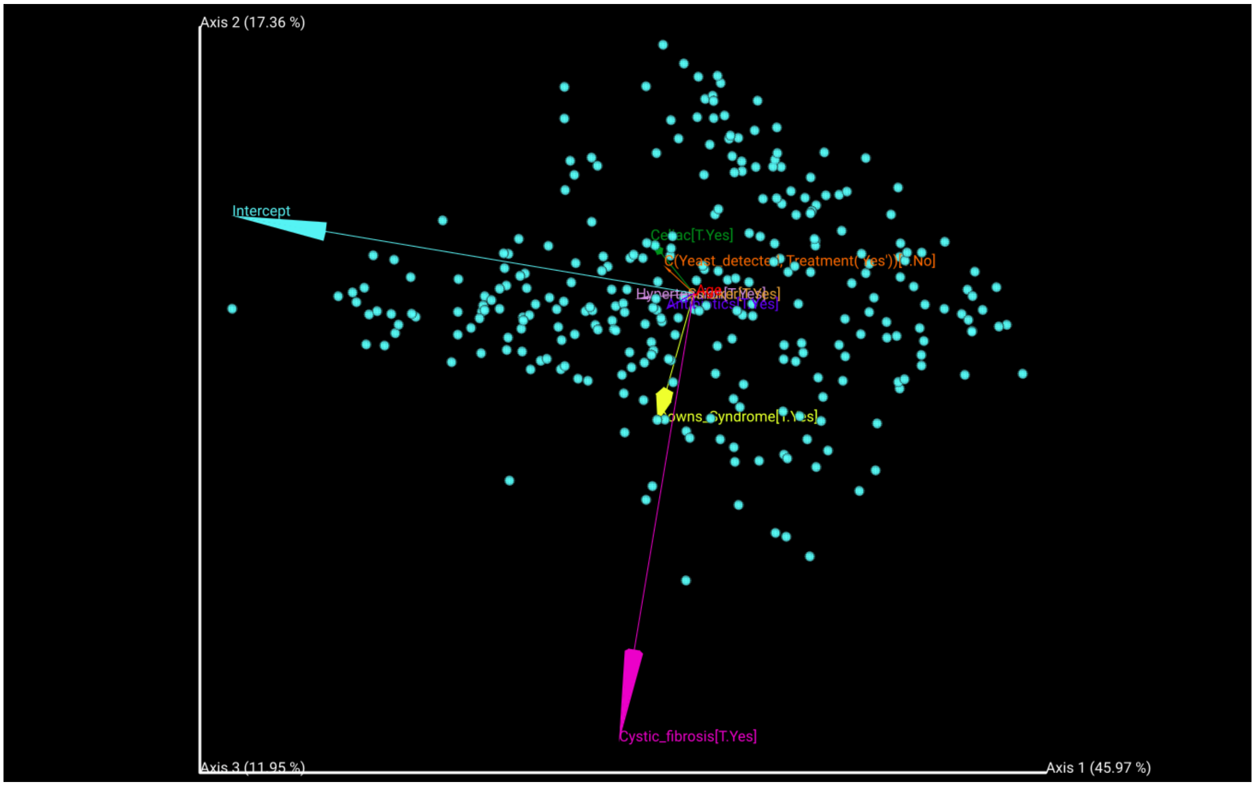

Supplementary Figure 2: Biplot from the emperor visualization tool and based on calculations using the songbird multinomial tool. Includes the variables from **Figure 2**, as well as Age as a continuous value, which gives support for the results in that figure and the conclusions drawn from it. Cystic fibrosis and Down Syndrome are the most impactful variables, celiac disease and hypertension are notably less significant, though still more so than the others, with the exception of the detection of yeast, which in this biplot is of a similar magnitude with those two disorders. This is the only discernible discrepancy, though minor. Meanwhile, age seems to be the least important of these variables, which supports our findings because this biplot is modeling the impact of a linear change across age. We showed that there is instead a sort of parabolic effect with age (**Figure 1**), where the youngest and oldest samples were the most variable and distinct, while the middle ages were relatively homogeneous.

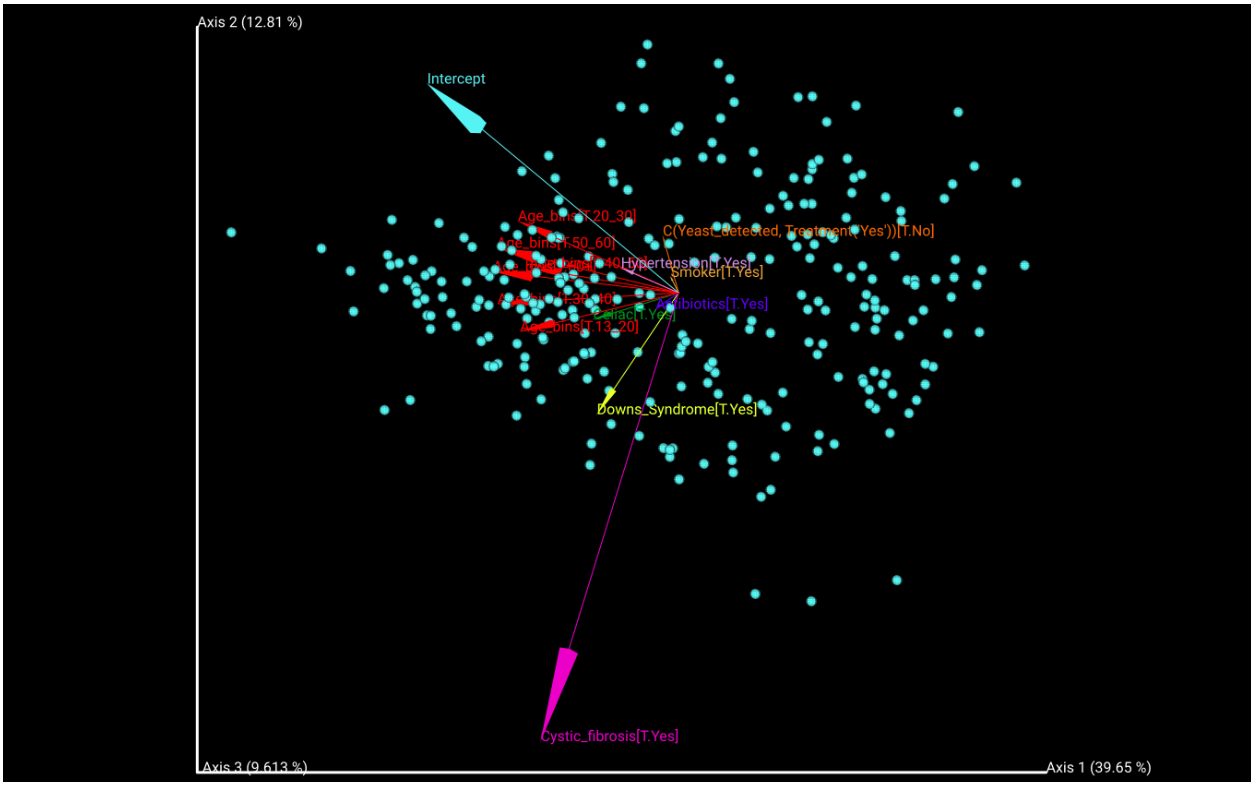

Supplementary Figure 3: Biplot from the emperor visualization tool and based on calculations using the songbird multinomial tool. Includes the variables from **Figure 2**, as well as the six Age bins instead of the continuous value of ages. Here it can be seen that, while cystic fibrosis is still the most impactful variable, each age bin has a somewhat distinct effect, and each is generally of a magnitude similar to that of Down Syndrome.

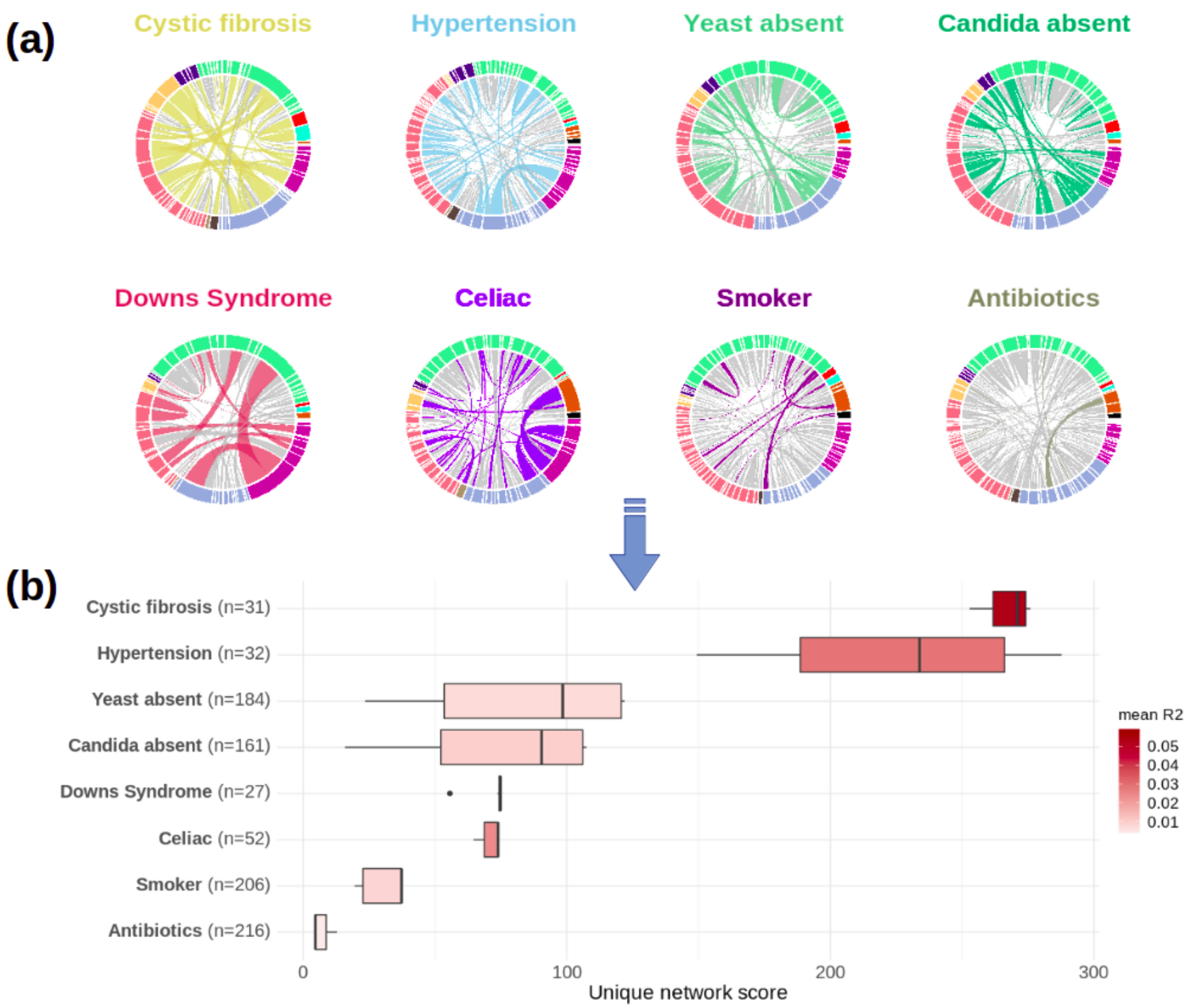

Supplementary Figure 4: Uniqueness of core co-occurrence networks. (a) Chord diagram plots displaying significant co-occurrences and their relative strengths among the indicated samples for a given variable. Line widths are proportional to the absolute values of the strengths of the associations between genera, which can be either positive or negative. Colored lines represent associations that were unique to the samples indicated for a variable when compared to the network of at least one other variable. Gray lines are significant associations that were not unique to any of the displayed variables. Segments of the outer rings represent genera and are colored by phyla. These are not labeled in a legend since this serves primarily as a schematic segue into the next part of the figure. **(b)** Boxes represent the distributions of scores derived from these unique associations. The values in a given box are the scores for that variable compared to each of the others. Thus the scores are relative only to these eight variables presented here.

Scores were calculated as follows: for each variable, the co-occurrence networks were calculated among each of the 100 subsamples, and we retained those associations which occurred only in the groups of interest (samples with the indicated disorder, smokers, antibiotic users, or those samples in which yeast was absent). Then for each variable, we calculated the number of only those associations which occurred in all 100 subsamples and in 0 subsamples of the other variable being compared, weighted by the absolute values of the strengths of those associations. Boxes are colored based on the mean R^2 value from the PERMANOVA tests comparing groups of a given variable. The number of samples for which a given variable was indicated in each subsample is indicated in the y axis.

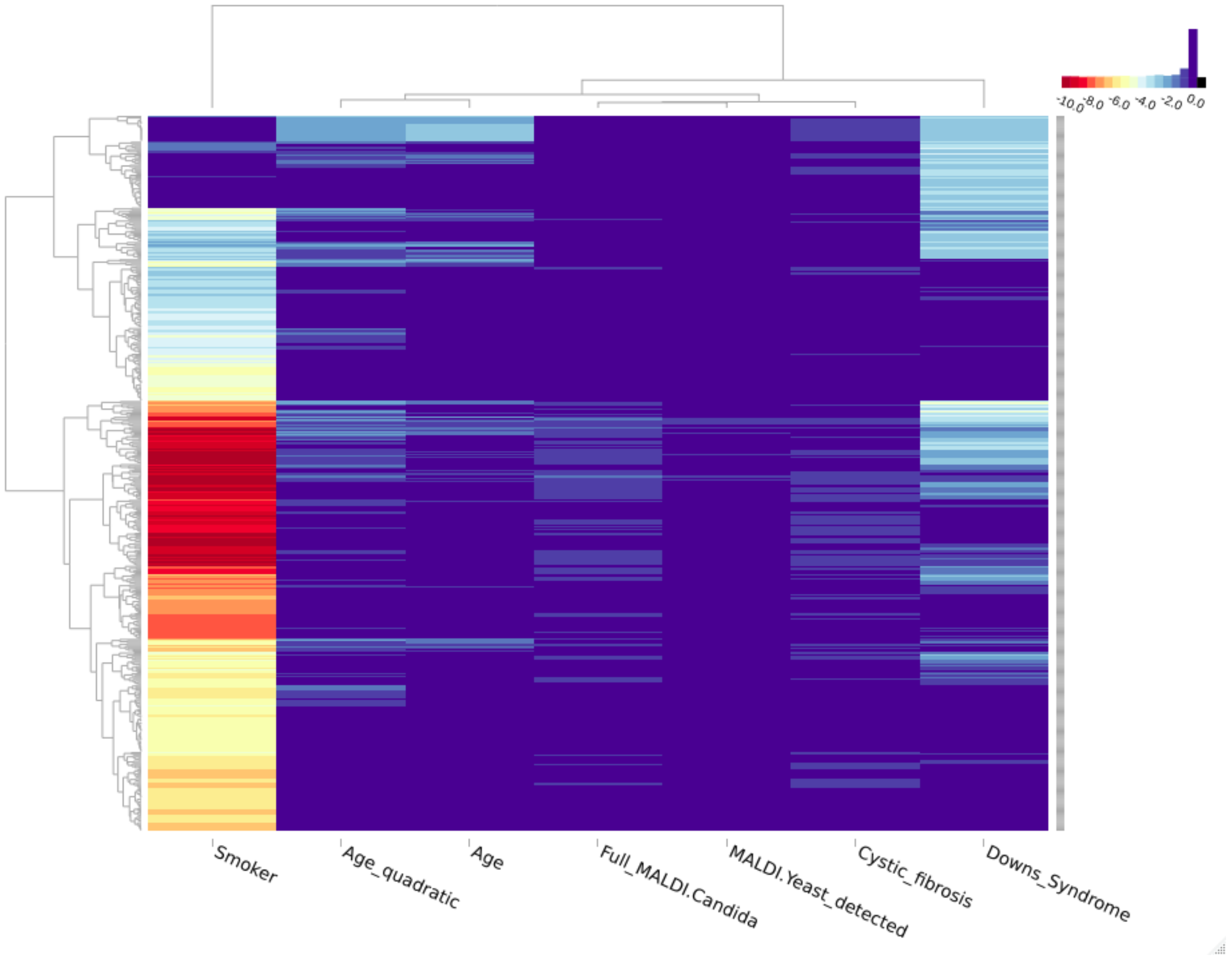

Supplementary Figure 5: Heatmap of significance of differential abundances of KEGG orthologs. The heatmap shows the log of the adjusted p-values from ANOVAs for tests of differential abundance of KOs in each variable. The variables shown were those that had at least one adjusted p-value less than 1.0, though smoker and Down Syndrome were the only variables with any significantly differentially abundant KOs (adjusted p-value < 0.05).

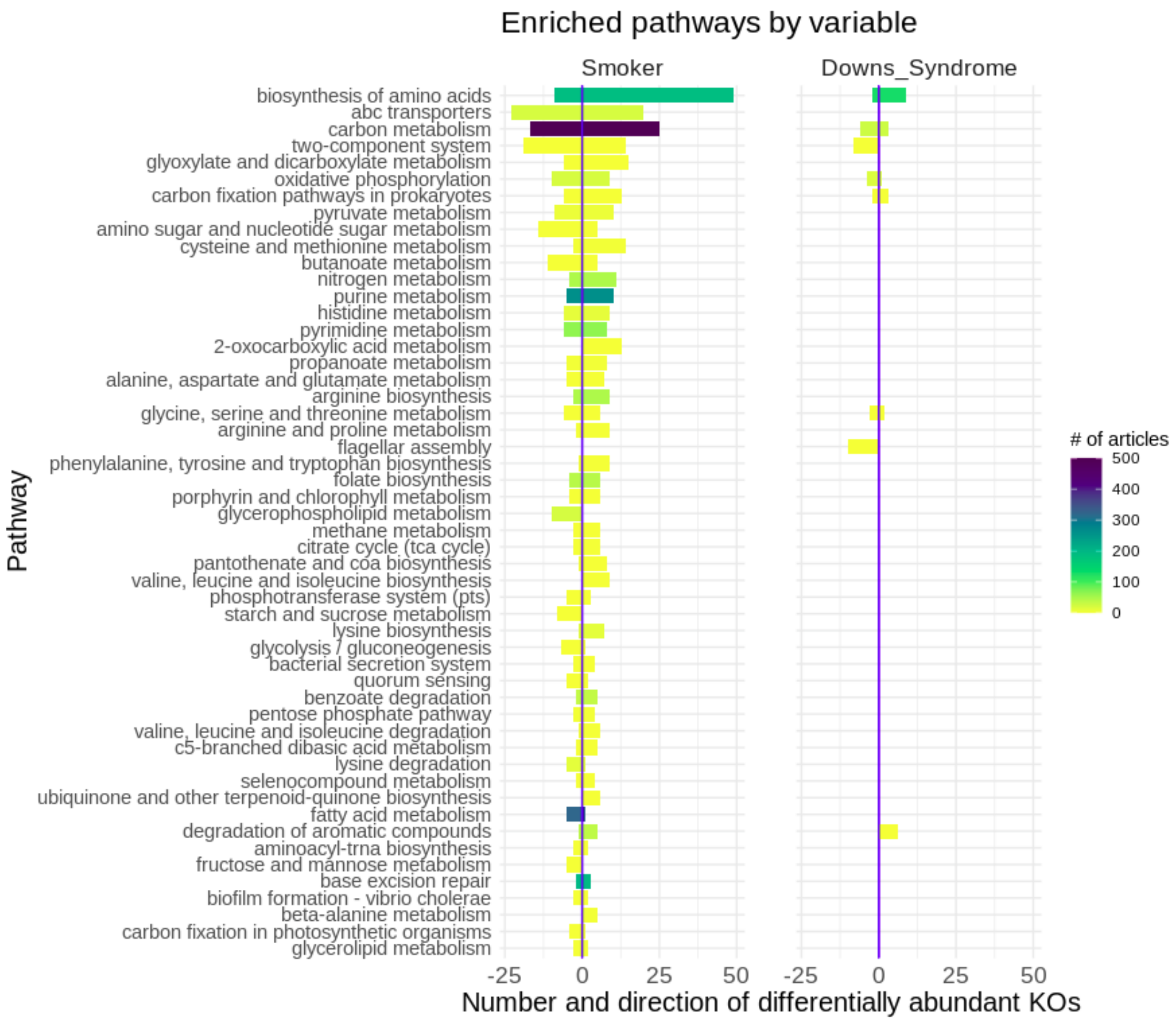

Supplementary Figure 6: Enriched pathways in smoking and Down Syndrome. Barplots for only those pathways with at least 5 significant KOs for a given variable. The x-axis indicates the number of KOs that differed both positively and negatively, wherein a positive value indicates an increase in that KO in smokers/DS and a negative value indicates a decrease in that KO in smokers/DS. The bars are shaded by the number of articles found to link a given pathway with either smoking or Down Syndrome using a text-mining approach.

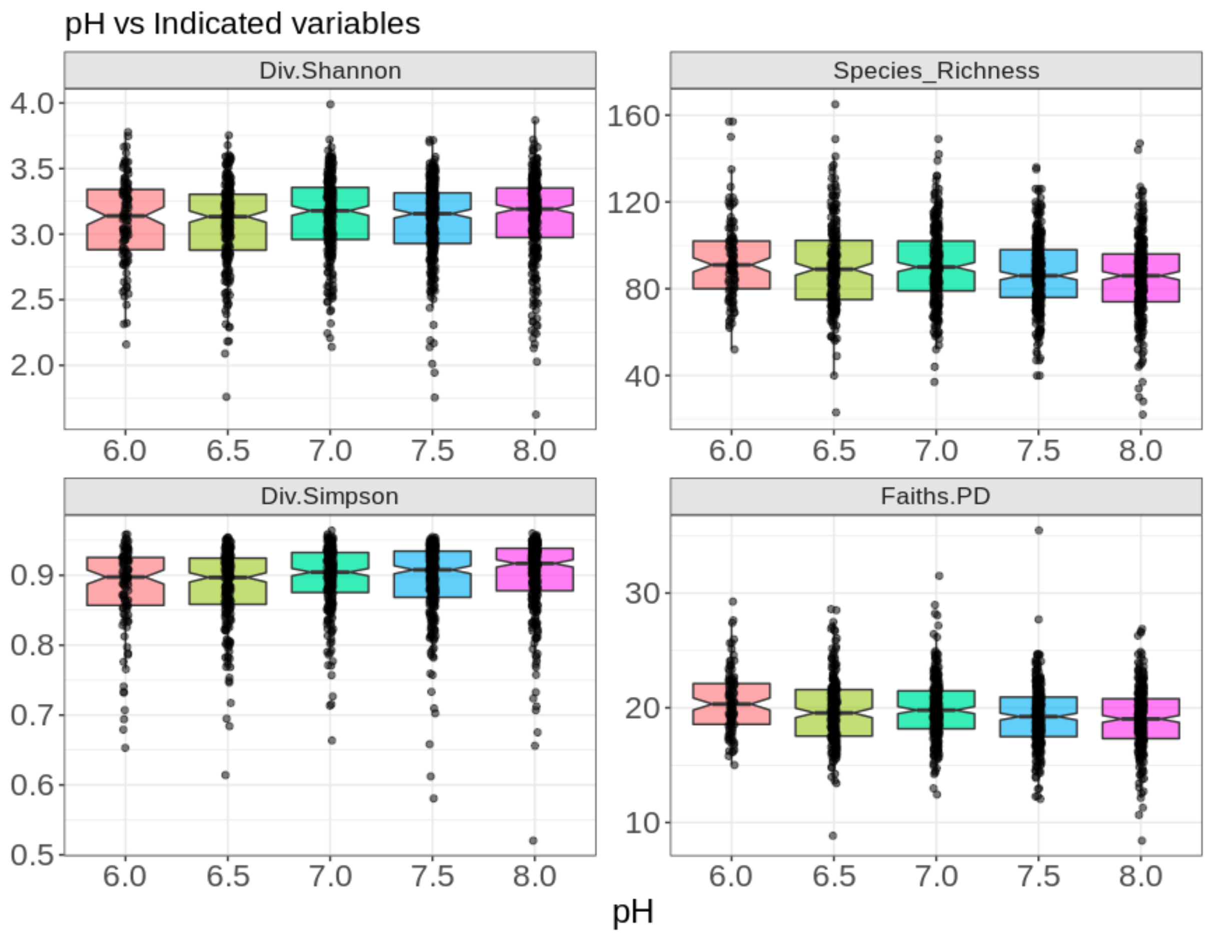

Supplementary Figure 7: Alpha diversity trends with pH. Boxplots show the distributions of four measures of alpha diversity at the different levels of pH measured in the oral cavities of the 1337 samples with no chronic disorders. As pH increased, Faith's phylogenetic diversity ($p = 2.97e-5$) and species richness ($p = 6.23e-5$) decreased, while there was no significant trend with either Shannon or Simpson diversity.

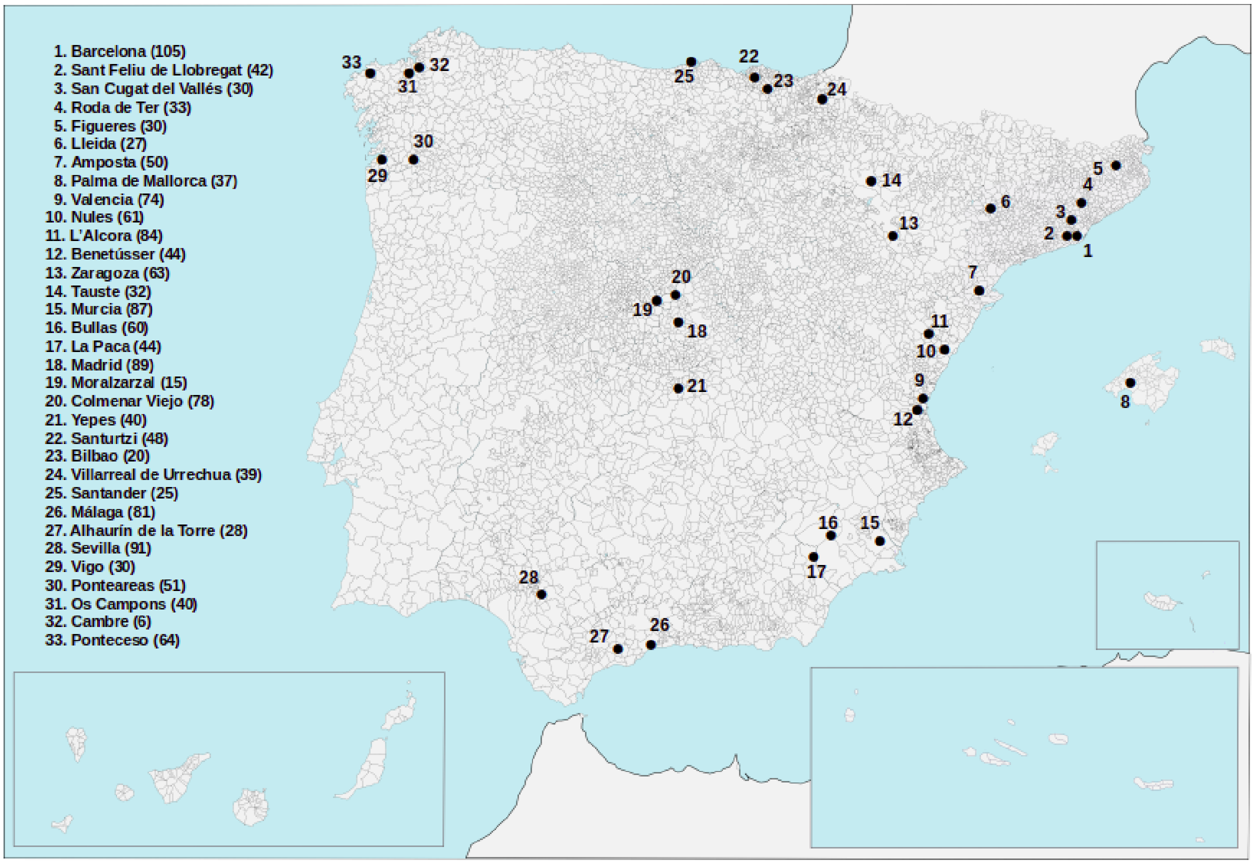

Supplementary Figure 8: Sample collection sites. Samples were collected from 60 different sites in 33 cities across Spain. This figure shows the locations of the cities from which the samples were collected, with the corresponding names listed on the left and the number of samples next to it. Map of Spain was obtained from Wikimedia Commons, P.d V, CC BY-SA 4.0 <https://commons.wikimedia.org/w/index.php?curid=88248164>.

Supplementary Table 1: Associations between taxa and pH. Columns indicate, in this order, the organism name, the tendency of the association with pH ("↗": abundance increases as pH in the oral cavity increases, "↘": abundance decreases as pH increases), the adjusted p-value of the ANOVA in the statistical comparison between the organism and pH, whether the organism has been found in the literature to be acidogenic ("+" indicates yes, "-" indicates no, and an empty cell indicates a lack of information), whether the organism has been found in the literature to be associated with dental caries, and references to the relevant literature.

Organism	With pH	Adj. p-value	Acidogenic	Dental caries	References
Porphyromonas	↗	1.19e-16	+	+	1
Bergeyella	↗	1.06e-5	-	-	2,3
Haemophilus	↗	4.10e-5		-	2
Johnsonella	↗	5.97e-5	-		4
Gemella	↗	1.59e-4	+	+/-	5–7
Alloprevotella	↗	5.34e-4	+		8
Streptobacillus	↗	6.79e-4	+		9
Peptostreptococcus	↗	0.00783	+		10
Actinobacillus	↗	0.00704		-	2
Abiotrophia	↗	0.00877	+	-	2
Olsenella	↘	4.10e-4		+	11
Megasphaera	↘	7.83e-4	-	-	12
Dialister	↘	0.00640		+	2,11
Selenomonas	↘	0.00704		+	1,2,13
Lactobacillus	↘	0.0217	+	+	2,14,15
Anaeroglobus	↘	0.0364		+	2,11

References

1. Radaic, A. & Kapila, Y. L. The oralome and its dysbiosis: New insights into oral microbiome-host interactions. *Comput. Struct. Biotechnol. J.* **19**, 1335–1360 (2021).
2. Wang, Y. *et al.* Profiling of Oral Microbiota in Early Childhood Caries Using Single-Molecule Real-Time Sequencing. *Front. Microbiol.* **8**, 2244 (2017).
3. Vandamme, P., Bernardet, J.-F., Segers, P., Kersters, K. & Holmes, B. NOTES: New Perspectives in the Classification of the Flavobacteria: Description of Chryseobacterium gen. nov., Bergeyella gen. nov., and Empedobacter nom. rev. *Int. J. Syst. Bacteriol.* **44**, 827–831 (1994).
4. Moore, L. V. & Moore, W. E. Oribaculum catoniae gen. nov., sp. nov.; Catonella morbi gen. nov., sp. nov.; Hallella seregens gen. nov., sp. nov.; Johnsonella ignava gen. nov., sp. nov.; and Dialister pneumosintes gen. nov., comb. nov., nom. rev., Anaerobic gram-negative bacilli from the human gingival crevice. *Int. J. Syst. Bacteriol.* **44**, 187–192 (1994).
5. Kilpper-Balz, R. & Schleifer, K. H. Transfer of Streptococcus morbillorum to the Genus Gemella as Gemella morbillorum comb. nov. *Int. J. Syst. Bacteriol.* **38**, 442–443 (1988).
6. Aas, J. A. *et al.* Bacteria of dental caries in primary and permanent teeth in children and young adults. *J. Clin. Microbiol.* **46**, 1407–1417 (2008).
7. Li, Y. *et al.* Oral microbial community typing of caries and pigment in primary dentition. *BMC Genomics* **17**, 558 (2016).
8. Downes, J., Dewhirst, F. E., Tanner, A. C. R. & Wade, W. G. Description of Alloprevotella rava gen. nov., sp. nov., isolated from the human oral cavity, and reclassification of Prevotella tannerae Moore et al. 1994 as Alloprevotella tannerae gen. nov., comb. nov. *Int. J. Syst. Evol. Microbiol.* **63**, 1214–1218 (2013).
9. Nolan, M. *et al.* Complete genome sequence of Streptobacillus moniliformis type strain (9901). *Stand. Genomic Sci.* **1**, 300–307 (2009).
10. Riggio, M. P. & Lennon, A. Development of a PCR assay specific for Peptostreptococcus anaerobius. *J. Med. Microbiol.* **51**, 1097–1101 (2002).
11. Wang, Y. *et al.* Oral Microbiome Alterations Associated with Early Childhood Caries Highlight the Importance of Carbohydrate Metabolic Activities. *mSystems* **4**, (2019).
12. Nallabelli, N. *et al.* Biochemical and genome sequence analyses of Megasphaera sp. strain DISK18 from dental plaque of a healthy individual reveals commensal lifestyle. *Sci. Rep.* **6**, 33665 (2016).
13. Esberg, A., Haworth, S., Hasslöf, P., Lif Holgerson, P. & Johansson, I. Oral Microbiota

Profile Associates with Sugar Intake and Taste Preference Genes. *Nutrients* **12**, (2020).

14. Caufield, P. W., Schön, C. N., Saraithong, P., Li, Y. & Argimón, S. Oral Lactobacilli and Dental Caries: A Model for Niche Adaptation in Humans. *J. Dent. Res.* **94**, 110S–8S (2015).
15. Georgios, A., Vassiliki, T. & Sotirios, K. Acidogenicity and acidurance of dental plaque and saliva sediment from adults in relation to caries activity and chlorhexidine exposure. *J. Oral Microbiol.* **7**, 26197 (2015).
