# Supplementary material for: Citizen-science reveals changes in the oral microbiome in Spain through age and lifestyle factors
Source: NPJ Biofilms Microbiomes. 2022 May 19;8:38. doi: 10.1038/s41522-022-00279-y (PMC9117221; doi:10.1038/s41522-022-00279-y)
Supplement: Supplementary file 2 — Reporting Summary Checklist [file 41522_2022_279_MOESM2_ESM.pdf]

nature portfolio

Corresponding author(s): Toni Gabaldón

Last updated by author(s): Jan 11, 2022

Reporting Summary

Nature Portfolio wishes to improve the reproducibility of the work that we publish. This form provides structure for consistency and transparency in reporting. For further information on Nature Portfolio policies, see our Editorial Policies and the Editorial Policy Checklist.

Statistics

For all statistical analyses, confirm that the following items are present in the figure legend, table legend, main text, or Methods section.

n/a	Confirmed	
☐	☒	The exact sample size (*n*) for each experimental group/condition, given as a discrete number and unit of measurement
☐	☒	A statement on whether measurements were taken from distinct samples or whether the same sample was measured repeatedly
☐	☒	The statistical test(s) used AND whether they are one- or two-sided<br>*Only common tests should be described solely by name; describe more complex techniques in the Methods section.*
☐	☒	A description of all covariates tested
☐	☒	A description of any assumptions or corrections, such as tests of normality and adjustment for multiple comparisons
☐	☒	A full description of the statistical parameters including central tendency (e.g. means) or other basic estimates (e.g. regression coefficient) AND variation (e.g. standard deviation) or associated estimates of uncertainty (e.g. confidence intervals)
☒	☐	For null hypothesis testing, the test statistic (e.g. *F*, *t*, *r*) with confidence intervals, effect sizes, degrees of freedom and *P* value noted<br>*Give P values as exact values whenever suitable.*
☒	☐	For Bayesian analysis, information on the choice of priors and Markov chain Monte Carlo settings
☒	☐	For hierarchical and complex designs, identification of the appropriate level for tests and full reporting of outcomes
☒	☐	Estimates of effect sizes (e.g. Cohen's *d*, Pearson's *r*), indicating how they were calculated

Our web collection on statistics for biologists contains articles on many of the points above.

Software and code

Policy information about availability of computer code

Data collection	*Provide a description of all commercial, open source and custom code used to collect the data in this study, specifying the version used OR state that no software was used.*
Data analysis	All statistical analyses were performed, and figures were produced, using custom R scripts (R version 3.6.2)

For manuscripts utilizing custom algorithms or software that are central to the research but not yet described in published literature, software must be made available to editors and reviewers. We strongly encourage code deposition in a community repository (e.g. GitHub). See the Nature Portfolio guidelines for submitting code & software for further information.

Data

Policy information about availability of data

All manuscripts must include a data availability statement. This statement should provide the following information, where applicable:

- Accession codes, unique identifiers, or web links for publicly available datasets
- A description of any restrictions on data availability
- For clinical datasets or third party data, please ensure that the statement adheres to our policy

The fastq files for the paired forward and reverse reads of the 16S rRNA sequencing of the 1,648 oral rinse samples used for the analyses in this study (57,221 Mb) were uploaded to the Sequence Read Archive (SRA) with the BioProject accession number PRJNA667146 and can be found here: http://www.ncbi.nlm.nih.gov/bioproject/667146. We also provide a table with the results of MALDI-TOF analyses of fungal composition (44 kb), which can be found here: https://github.com/Gabaldonlab/ngs_public/tree/master/SLL2. The unique and anonymized identifiers for each sample can be found at the beginning of each fastq file, and these correspond to the row names in the fungal composition tables.

March 2021

Field-specific reporting

Please select the one below that is the best fit for your research. If you are not sure, read the appropriate sections before making your selection.

☒ Life sciences ☐ Behavioural & social sciences ☐ Ecological, evolutionary & environmental sciences

For a reference copy of the document with all sections, see nature.com/documents/nr-reporting-summary-flat.pdf

Life sciences study design

All studies must disclose on these points even when the disclosure is negative.

Sample size	We collected 1648 oral rinse samples from cities all across Spain, using a citizen science-based study design, wherein samples were collected on a voluntary basis.
Data exclusions	No data were excluded.
Replication	Where appropriate, we ran all statistical tests in randomized sub-samples in order to ensure the reproducibility of results.
Randomization	Sub-samples were randomized and used in statistical tests only when the relevant groups within those sub-samples were shown to maintain relative balances of age range, gender, and geographical location, as described in the methods section.
Blinding	This was an exploratory study, and as such, there were no treatment groups requiring blinding, outside of the randomization of sub-samples for statistical tests.

Reporting for specific materials, systems and methods

We require information from authors about some types of materials, experimental systems and methods used in many studies. Here, indicate whether each material, system or method listed is relevant to your study. If you are not sure if a list item applies to your research, read the appropriate section before selecting a response.

Materials & experimental systems

n/a	Involved in the study	
☒	☐	Antibodies
☒	☐	Eukaryotic cell lines
☒	☐	Palaeontology and archaeology
☒	☐	Animals and other organisms
☐	☒	Human research participants
☒	☐	Clinical data
☒	☐	Dual use research of concern

Methods

n/a	Involved in the study	
☒	☐	ChIP-seq
☒	☐	Flow cytometry
☒	☐	MRI-based neuroimaging

Human research participants

Policy information about studies involving human research participants

Population characteristics	All samples were collected from participants within Spain, and included ages ranging from 7 to 85 years old, 937 females and 709 males, and subsets of participants with particular chronic disorders, including cystic fibrosis (n=31), Down Syndrome (n=27), and celiac disease (n=52).
Recruitment	For part of the sample set, a team member went to high schools across Spain to collect from students, as well as some of their teachers and family members. In addition, our team contacted a number of national associations for particular chronic disorders that we wished to study, as described in the manuscript.
Ethics oversight	This project was approved by the ethics committee of the Barcelona Biomedical Research Park (PRBB)

Note that full information on the approval of the study protocol must also be provided in the manuscript.
